# MHEALTH DESIGN AND IMPLEMENTATION IMPLICATIONS TO PROMOTE PHYSICAL ACTIVITY IN OLDER HISPANICS/LATINOS WITH MCI

**DOI:** 10.1093/geroni/igac059.687

**Published:** 2022-12-20

**Authors:** G Adriana Perez

**Affiliations:** University of Pennsylvania, Philadelphia, Pennsylvania, United States

## Abstract

Addressing physical inactivity for older Hispanics/Latinos with mild cognitive impairment (MCI) is a public health priority, since MCI increases the risk of developing Alzheimer’s Disease and related dementias (ADRD). Compared to non-Latino Whites, Hispanics/Latinos are 2X more likely to develop ADRD. One promising approach to promoting physical activity includes use of mobile health (mHealth) strategies that may deliver Spanish-language information and culturally relevant motivational messages to enhance intrapersonal/interpersonal factors for health behavior change. The purpose of this study is to test mHealth strategies as a mechanism to deliver booster sessions for reinforcing physical activity goals/progress among older Latinos with MCI who complete the Tiempos Juntos intervention treatment. Our hypothesis is that culturally adapting mHealth strategies may improve efficacy and maintenance of physical activity effect; as well as cognitive health outcomes (6 months post-intervention). Results among the first wave of participants will be discussed, including challenges and opportunities for future research. While the use of mHealth is not new, these approaches commonly exclude individuals with limited-English proficiency and thus, designing interventions that center the needs of older Hispanics/Latinos with MCI/ADRD and other historically excluded communities, is an important first step in promoting physical activity and advancing cognitive health equity.

